# A novel spliced fusion of *MLL *with *CT45A2 *in a pediatric biphenotypic acute leukemia

**DOI:** 10.1186/1471-2407-10-518

**Published:** 2010-09-29

**Authors:** Nuno Cerveira, Claus Meyer, Joana Santos, Lurdes Torres, Susana Lisboa, Manuela Pinheiro, Susana Bizarro, Cecília Correia, Lucília Norton, Rolf Marschalek, Manuel R Teixeira

**Affiliations:** 1 Department of Genetics of the Portuguese Oncology Institute, Porto, Portugal; 2 Cancer Genetics Group, Research Centre of the Portuguese Oncology Institute, Porto, Portugal; 3 Institute of Pharmaceutical Biology, Diagnostic Centre of Acute Leukemia (DCAL), Goethe-University of Frankfurt, Frankfurt/Main, Germany; 4 Department of Pediatrics, Portuguese Oncology Institute, Porto, Portugal; 5 Biomedical Sciences Institute (ICBAS), Porto, Portugal

## Abstract

**Background:**

Abnormalities of 11q23 involving the *MLL *gene are found in approximately 10% of human leukemias. To date, nearly 100 different chromosome bands have been described in rearrangements involving 11q23 and 64 fusion genes have been cloned and characterized at the molecular level. In this work we present the identification of a novel *MLL *fusion partner in a pediatric patient with *de novo *biphenotypic acute leukemia.

**Methods:**

Cytogenetics, fluorescence in situ hybridization (FISH), molecular studies (RT-PCR and LDI-PCR), and bioinformatic sequence analysis were used to characterize the *CT45A2 *gene as novel *MLL *fusion partner in pediatric acute leukemia.

**Results:**

Fluorescence *in situ *hybridization of bone marrow G-banded metaphases demonstrated a cryptic insertion of 11q23 in Xq26.3 involving the *MLL *gene. Breakpoint fusion analysis revealed that a DNA fragment of 653 kb from 11q23, containing *MLL *exons 1-9 in addition to 16 other 11q23 genes, was inserted into the upstream region of the *CT45A2 *gene located at Xq26.3. In addition, a deletion at Xq26.3 encompassing the 3' region of the *DDX26B *gene (exons 9-16) and the entire *CT45A1 *gene was identified. RNA analysis revealed the presence of a novel *MLL-CT45A2 *fusion transcript in which the first 9 exons of the *MLL *gene were fused in-frame to exon 2 of the *CT45A2 *gene, resulting in a spliced *MLL *fusion transcript with an intact open reading frame. The resulting chimeric transcript predicts a fusion protein where the N-terminus of MLL is fused to the entire open reading frame of CT45A2. Finally, we demonstrate that all breakpoint regions are rich in long repetitive motifs, namely LINE/L1 and SINE/Alu sequences, but all breakpoints were exclusively identified outside these repetitive DNA sequences.

**Conclusion:**

We have identified *CT45A2 *as a novel spliced *MLL *fusion partner in a pediatric patient with *de novo *biphenotypic acute leukemia, as a result of a cryptic insertion of 11q23 in Xq26.3. Since *CT45A2 *is the first Cancer/Testis antigen family gene found fused with *MLL *in acute leukemia, future studies addressing its biologic relevance for leukemogenesis are warranted.

## Background

Abnormalities of 11q23 involving the *MLL *gene are found in approximately 10% of human leukemias [1]. *MLL *rearrangements are present in >70% of infant leukemias, irrespective of the immunophenotype being more consistent with acute lymphoblastic leukemia (ALL) or acute myeloid leukemia (AML), but are less frequent in leukemias from older children [2]. *MLL *translocations are also found in approximately 10% of adult AML, and can also be found in a proportion of patients with therapy-related leukemia after treatment for other malignancies with topoisomerase II inhibitors [3]. Although clinically and morphologically heterogeneous, *MLL*-rearranged ALL and AML show unique gene expression profiles [4,5].

To date, nearly 100 different chromosome bands have been described in rearrangements involving 11q23 and 64 fusion genes have been cloned and characterized at the molecular level [6]. The most common *MLL *fusion partners are *AFF1/AF4 *(4q21), *MLLT3/AF9 *(9p23), *MLLT1/ENL *(19p13.3), *MLLT10/AF10 *(10p12), *MLLT4/AF6 *(6q27), *ELL *(19p13.1), *EPS15/AF1P *(1p32), *MLLT6/AF17 *(17q21), and *SEPT6 *(Xq24) [6]. Usually, *MLL *rearrangements result from the non-homologous-end-joining (NHEJ) DNA repair pathway following DNA damage [7]. Reciprocal chromosomal translocations are the most frequent events associated with the genetic recombination of *MLL*, but other mechanisms have been identified, including internal partial tandem duplication (*MLL*-PTD), chromosome 11 deletions or inversions, and several types of complex *MLL *rearrangements [6]. Occasionally, chromosomal translocation or deletion have been described to originate *MLL *spliced fusions, which arise by fusing the 5' *MLL *region to downstream located partner genes [8]. In the present study, we have identified the *CT45A2 *gene as a novel fusion partner of *MLL *in a pediatric patient with *de novo *biphenotypic acute leukemia (BAL), as a result of a cryptic insertion of 11q23 material in Xq26 resulting in a spliced *MLL *fusion.

## Methods

### Patient Data

A 6-year-old boy was admitted to the Portuguese Oncology Institute (Porto, Portugal) with a history of fever, asthenia and cutaneous pallor. Peripheral blood analysis revealed anemia (Hb 6.3 g/dl) and bicytopenia. Bone marrow analysis revealed the presence of 51% of blasts with the immunophenotype CD3+, CD13+, CD33+, and CD117+, which lead to the diagnosis of biphenotypic phenotype (T/myeloid) acute leukemia. No blasts were detected in the cerebrospinal fluid. He was treated according to the ELAM 02 protocol (aracytine, mitoxantrone and methotrexate) and entered complete remission after induction chemotherapy. Seven months later he was submitted to allogeneic bone marrow transplantation with umbilical cord hematopoietic progenitors, but the patient showed evidence of relapse after one year. Treatment with the AML relapse protocol was started, but only partial remission had been achieved four months later. The patient underwent a haploidentical transplant with his mother's peripheral blood cell progenitors, but the disease relapsed again and the patient died nine months later.

### Chromosome Banding and Molecular Cytogenetics

The diagnostic bone marrow sample was cultured for 24 hours in RPMI 1640 medium with GlutaMAX-I (Invitrogen, London, UK) supplemented with 20% fetal bovine serum (Invitrogen, London, UK). Chromosome preparations were made by standard methods and banded by trypsin-Leishman. Karyotypes were described according to the International System for Human Cytogenetic Nomenclature [9]. Fluorescence in situ hybridization (FISH) analysis was performed using the LSI MLL Dual-Color, Break-Apart Probe and LSI IGH/BCL2 Dual-Color, Dual Fusion Probe (Vysis, Downers Grove, USA), respectively, in previously stained metaphases that have been destained and processed for FISH as described [10].

### RNA and DNA Extraction

High molecular weight DNA and RNA were extracted from the bone marrow sample using 1 ml of Tripure isolation reagent (Roche Diagnostics, Indianapolis, USA), according to the manufacturer's instructions.

### Long-Distance-Inverse Polymerase Chain Reaction (LDI-PCR)

The DNA sample was treated and analyzed as previously described [11,12]. Briefly, 1 μg of genomic DNA was digested with restriction enzymes and self-ligated to form DNA circles. Amplification was performed with specific primers for fusion sequences on der(11) and der(X). LDI-PCR reactions were performed as described [12] and according to the manufacturer's recommendations (PCR Extender System, 5 Prime, Hamburg, Germany). Amplified products were analyzed on a 1% agarose gel (SeaKem LE Agarose, Rockland, USA). PCR amplimers were isolated from the gel and subjected to DNA sequence analyses to obtain the patient-specific fusion sequences. After sequencing, unknown sequences were characterized by blasting the human genome database (Genomic BLAST, http://blast.ncbi.nlm.nih.gov/Blast.cgi).

### Reverse-Transcription Polymerase Chain-Reaction (RT-PCR)

For cDNA synthesis, 1 μg of RNA was subjected to reverse transcription with random hexamers using the Superscript III First-Strand Synthesis System for RT-PCR (Invitrogen, Carlsbad, USA), according to the manufacturer's instructions. RT-PCR assay for detection of *MLL-CT45A2 *fusion transcripts was performed with a forward primer (MLL-S; 5'-GAGGATCCTGCCCCAAAGAAAAG-3') located in *MLL *exon 8 (GenBank accession no. NM_005933) and a reverse primer (CT45A2-AS; 5'- GGCCATCCTCTGCCTTTTC-3') located in *CT45A2 *exon 2 (GenBank accession no. NM_152582). Additional primers in the *MLL *breakpoint cluster region (exons 9 to 13) and *CT45A2 *open reading frame (exons 3 to 5) were used to exclude the presence of additional splice variants (data not shown). PCR reactions were performed in a 50 μl reaction volume containing 2 μl of synthesized cDNA, 5 μl of 10× GeneAmp PCR buffer II (100 mM Tris-HCl pH 8.3, 500 mM KCl) (Applied Biosystems, Foster City, USA), 5 μl of 25 mM MgCl_2_, 0.4 μl dNTP mix (25 mM each dNTP) (Applied Biosystems), 0.4 mM of each primer (Metabion, Martinsried, Germany), and 1 unit of AmpliTaq Gold DNA Polymerase (Applied Biosystems). Reaction tubes were kept on ice at all times to prevent non-specific amplification and incubated for 5 min at 94°C, followed by 35 cycles of 30 sec. at 95°C, 1 min at 63°C, and 1.5 min at 72°C, followed by a final elongation of 10 min at 72°C on a GeneAmp PCR System 9700 (Applied Biosystems). Amplified products were analyzed on a 2% agarose gel (SeaKem LE Agarose) and the results were visualized in an image analyzer ImageMaster VDS (Amersham Biosciences, Little Chalfont, UK).

### Bioinformatic Sequence Analysis

Search for DNA sequence motifs known to be associated with site-specific recombination, mutation, cleavage, and gene rearrangement, and repetitive sequence elements spanning or in the vicinity of deletion and insertion breakpoints [13,14] was performed with SEQ tools [15] and RepeatMasker [16]. Search for repetitive sequence homology was performed with ClustalW [17].

## Results

### Karyotyping and Molecular Cytogenetics

**Figure 1:**
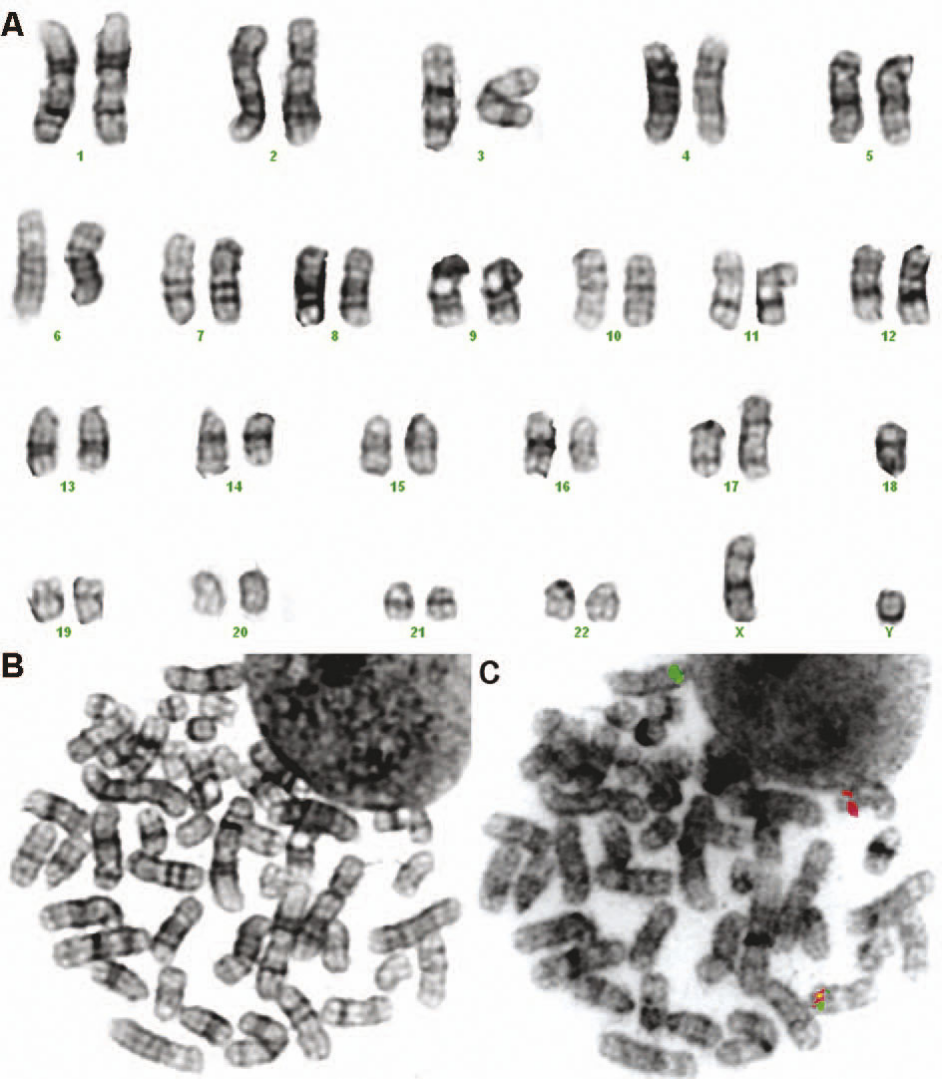
**Cytogenetic analysis of the bone marrow sample of the AML patient**. (A) Karyogram showing abnormalities of chromosomes 14 and 17 and monosomy of chromosome 18. The same metaphase after G-banding (B) and after destaining and processed for dual-color FISH analysis (C) showing a cryptic insertion of the 5' *MLL *gene region (green) in Xq22~25.

The bone marrow cytogenetic analysis revealed abnormalities of chromosomes 14 and 17 and monosomy of chromosome 18 (Figure 1A-B). FISH analysis on previously G-banded metaphases showed a cryptic insertion of the 5' *MLL *gene region in Xq22~25 (Figure 1C), which suggested a rearrangement of the *MLL *gene, and also the presence of a *BCL2 *gene copy on the der(17). Based on the chromosome banding and FISH findings, the karyotype was described as 45,XY,add(14)(q24),add(17)(p13),-18[8].ish ins(X;11)(q22~25;q23q23)(MLL5'+;MLL5'-,MLL3'+), add(14)(IGH-),der(17)(17qter→17p13::?::18q21→18q21::?)(BCL2+)/46,XY[12].

### Characterization of the 11q23 and Xq22~25 Genomic Breakpoints

**Figure 2:**
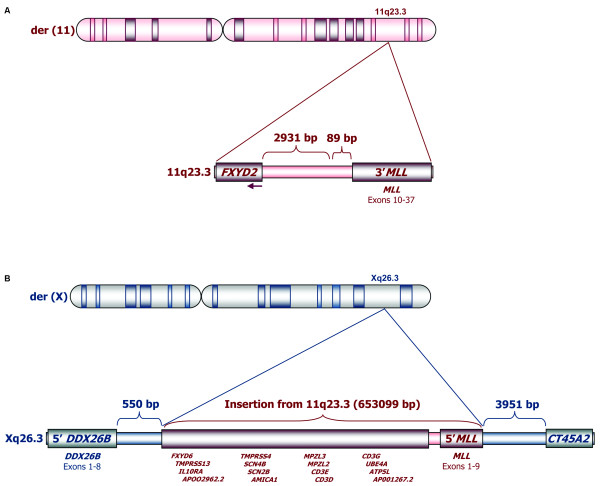
**Schematic representation of the cryptic insertion of 11q23 sequences in Xq26.3**. The 11q23 breaks occurred 2,931 bp downstream of the *FXYD2 *gene and 89 bp upstream of *MLL *exon 10, respectively (A). A large fragment of 653,099 bp from chromosome 11q23 was inserted in Xq26.3 (B), leading to the transfer to Xq26.3 not only of the 5' *MLL *region (exons 1 to 9) but also of 16 other genes (*FXYD6*, *TMPRSS13*, *IL10RA*, *APOO2962.2*, *TMPRSS4*, *SCN4B*, *SCN2B*, *AMICA1*, *MPZL3*, *MPZL2*, *CD3E*, *CD3D*, *CD3G*, *UBE4A*, *ATP5L*, and *AP001267.2*). The insertion resulted in the disruption of the *DDX26B *gene (550 bp downstream of *DDX26B *exon 8) and placed the 5' *MLL *region 3,951 bp upstream of the *CT45A2 *gene. As a result of the insertion, the *CT45A1 *gene was also deleted.

Using LDI-PCR as described above, the patient's sample was screened for germline and non-germline PCR amplimers that were subjected to sequence analysis. The results implicated that a large fragment of 653,099 bp from chromosome 11q23 was inserted into Xq26.3 (Figure 2). We determined that the 5' and 3' breaks in 11q23 occurred 2,931 bp downstream of the *FXYD2 *gene and 89 bp upstream of *MLL *exon 10, respectively, with 19 bp being deleted from *MLL *intron 9 (TGTTTTTTAGATCTATTAA) and with the insertion of two nucleotides at the breakpoint junction (GG; see Figure 2A). The insertion of 11q23 material into Xq26.3 contained the 5' *MLL *region (exons 1 to 9) in addition to 16 other 11q23 genes (*FXYD6*, *TMPRSS13*, *IL10RA*, *APOO2962.2*, *TMPRSS4*, *SCN4B*, *SCN2B*, *AMICA1*, *MPZL3*, *MPZL2*, *CD3E*, *CD3D*, *CD3G*, *UBE4A*, *ATP5L*, and *AP001267.2*; see Figure 2B). Regarding Xq26.3, we found that the insertion had occurred in *DDX26B *intron 8, 550 bp downstream of *DDX26B *exon 8 and 3,951 bp upstream of the *CT45A2 *gene, with the insertion of two (CG) and three additional nucleotides (GAA) at the breakpoint junctions, respectively (Figure 2B). In addition to the insertion, a large fragment of Xq26.3, encompassing the 3' region of *DDX26B *(exons 9-16) and the *CT45A1 *gene, was deleted.

The loss of 11q23 material led to an interstitial deletion on the der(11) chromosome. A more centromeric gene located at 11q23.3, *FXYD2*, was found to be localized upstream of *MLL *exons 10-37. However, the intact *FXYD2 *gene is oriented telomere to centromere, thus giving rise to a transcript into the opposing direction with regard to the orientation of the remaining 3'-*MLL *gene. Nevertheless, the 3'-*MLL *gene could possibly be transcribed from the recently identified gene internal promoter upstream of *MLL *exon 12 [18], resulting in 5'-truncated MLL protein fragment of about 230 kDa. However, transcripts starting directly at *MLL *intron11/exon12 borderline can hardly be distinguished from endogenous *MLL *transcripts deriving from the intact chromosome 11. Therefore, we investigated this not further.

### Characterization of MLL-CT45A2 Fusion Transcripts

**Figure 3:**
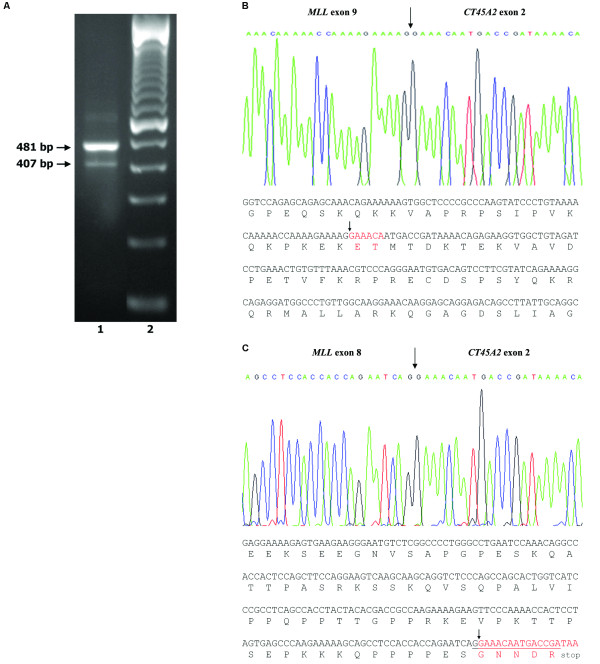
**Detection and analysis of the *MLL-CT45A2 *fusion transcript**. (A) RT-PCR analysis with a *MLL *sense primer located in *MLL *exon 8 and one *CT45A2 *sense primer located in *CT45A2 *exon 2 (lane 1). Lane 2 - 100 bp molecular marker. (B) Partial sequence of the junction of the *MLL-CT45A2 *e9e2 chimeric mRNA, showing the nucleotide sequence of the fusion transcript containing 6 bp (in red) from the 5' UTR of *CT45A2 *exon 2 coding for two additional amino acids. The arrow shows the in-frame fusion between *MLL *exon 9 and *CT45A2 *exon 2. (C) Partial sequence of the junction of the *MLL-CT45A2 *e8e2 chimeric mRNA, showing the nucleotide sequence of the fusion transcript. The arrow shows the out-of-frame fusion between *MLL *exon 8 and *CT45A2 *exon 2 that leads to a premature stop codon 14 nucleotides downstream of fusion breakpoint.

RT-PCR with an sense primer located on *MLL *exon 8 and an antisense primer located on *CT45A2 *exon 2 resulted in two PCR amplimers of 407 bp (weak) and 481 bp (strong), respectively (Figure 3A). Additional RT-PCR analysis with antisense primers located on *CT45A2 *exon 3 and a sense primer located on *MLL *exon 8 gave additional support to these results (data not shown). Sequencing of the 481 bp amplification product followed by a BLAST search confirmed that *MLL *exon 9 was fused in-frame with nucleotide 240 of the *CT45A2 *transcript (GenBank accession no. NM_152582; see Figure 3B). This fusion transcript contains 6 bp from the 5-UTR of *CT45A2 *exon 2 fortuitously coding for two additional amino acids (Figure 3, marked in red). The fainter 407 bp amplification product was also sequenced, and an out-of-frame splicing of *MLL *exon 8 to *CT45A2 *exon 2 was detected that leads to a premature stop codon 14 nucleotides downstream of the splice junction (Figure 3C). The in-frame fusion is predicted to give rise to a chimeric protein where the N-terminus of MLL is fused to the entire open reading frame of CT45A2. The putative MLL-CT45A2 fusion protein of 1,514 amino acids contains 1,325 amino acids from the N-terminal portion of MLL and 189 amino acids deriving from the CT45A2 protein.

### Search for DNA Recombination-Associated Motifs

**Figure 4:**
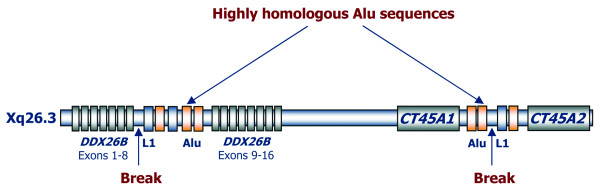
**Schematic representation of the 11q23.3 and Xq26.3 breakpoint regions**. The localization of the long repetitive sequences including the two highly homologous Alu sequences located in the vicinity of the Xq26.3 breakpoint is depicted. L1 (LINE/L1), L2 (LINE/L2), Alu (SINE/Alu), LCR (Low Complexity Repeat), MIR (SINE/MIR), ERVL (LTR/ERVL), MaLR (LTR/MaLR). None of the breakpoints were located inside these repetitive elements.

We searched all the breakpoint regions (2000 bp each side) for interspersed repeats and low complexity DNA sequences. All breakpoint regions were found to be rich in long repetitive sequences (Figure 4): *MLL *intron 9 (four SINE/Alu sequences), *DDX26B *intron 8 (three SINE/Alu sequences, two LINE/L1 sequences, two LINE/L2 sequences, and one SINE/MIR sequence), the 5'*CT45A2 *breakpoint region (three SINE/Alu sequences, one LINE/L1 sequence, four LTR/ERVL sequences, two LTR/MaLR sequences, and one low complexity repeat), and the 3'*FXYD2 *breakpoint region (two SINE/Alu sequences, two SINE/MIR sequences, and one low complexity repeat). Homology search showed that two of the Xq26.3 SINE/Alu sequences located 3' of the *DDX26B *breakpoint (2189-2492 bp) and 5' of the *CT45A2 *breakpoint (155-391 bp) where highly homologous (77%) (Figure 4). Moreover, we searched the vicinity of the breakpoint junctions (25 bp each side) for short repetitive sequences and found an inverted repeat (TTCACTT-AAGTGAAA) flanking the 5'*CT45A2 *breakpoint junction (-9 to -3 bp and +1 to +7 bp). However, all analyzed chromosomal breakpoints were always located outside of these repetitive DNA sequences, indicating that they are possibly not involved in the recombination event.

## Discussion

We have identified a novel fusion partner of *MLL*, the *CT45A2 *gene, which is a member of the Cancer/Testis (CT) gene family cluster localized at Xq26.3. In our pediatric patient with *de novo *biphenotypic acute leukemia, the *MLL-CT45A2 *fusion resulted from a cryptic insertion of 11q23 material into Xq26.3. The insertion of the 5'*MLL *region upstream of the *CT45A2 *gene leads to *MLL-CT45A2 *fusion transcripts. In our case, a weaker transcript exhibited *MLL *exons 1-8 fused to *CT45A2 *exon 2 and consecutive exons. This transcript is out-of-frame and produces only a truncated version of the MLL protein. The stronger PCR product, however, represented an in-frame fusion transcript containing *MLL *exons 1-9 fused to the intact *CT45A2 *transcript, a process known as spliced *MLL *fusion since the chimeric *MLL-CT45A2 *is only generated at the RNA level [8]. Interestingly, the chimeric mRNA contains 6 bp from the 5-UTR of *CT45A2 *exon 2 resulting in two additional amino acids. To our knowledge, this is the first description of a spliced *MLL *fusion resulting from an insertion event. Indeed, spliced *MLL *fusions were previously described in leukemia patients with the translocations t(1;11) (*MLL-EPS15*), t(4;11) (*MLL-AFF1*), t(9;11) (*MLL-MLLT3*), t(11;15) (*MLL-MPFYVE*), t(11;19) (*MLL-MLLT1*) and t(11;22) (*MLL-SEPT5*) [6,8,19], as well as in a single leukemia patient with an intrachromosomal 11q23 deletion (*MLL-DCPS*) [11]. Spliced *MLL *fusions can occur either by transcriptional read-through followed by a subsequent splice event or by trans-splicing [11]. Regardless of the underlying mechanism, the chimeric *MLL-CT45A2 *fusion is only produced at the RNA level. A reciprocal *CT45A2-MLL *transcript does not exist because the *FXYD2 *gene, transcribing in the opposite direction, is located upstream of the remaining *MLL *exons 10-37. However, we cannot exclude that the recently described gene-internal promoter upstream of *MLL *exon 12 is able to produce a 5'-truncated MLL protein of about 230 kDa [18].

Another consequence of this spliced fusion is that the expression of the *CT45A2 *gene, usually restricted to testicular tissue [20], is activated. CT genes encode a heterogeneous group of immunogenic proteins (CT antigens) that were initially identified as immunogenic tumor antigens and whose expression is almost restricted to the normal testis and a percentage of various tumor types, including melanoma and carcinomas of the bladder, lung and liver [20-24]. The combination of restricted normal tissue expression, spontaneous immunogenicity and frequent tumor expression has made these antigens attractive targets for cancer vaccines [21,24]. The CT45 gene family comprises six members (*CT45A1 *to *CT45A6*) located in Xq26.3 that are near-identical gene copies, suggesting the occurrence of recent gene duplications, but whose function remains to be elucidated [20]. The phenotypic consequences of *CT45A2 *expression in the leukemia cells of this patient are currently unknown. However, as CT45A2 exhibits the typical CT antigen immunogenic profile, it would be worthy to further investigate the possible humoral and cell-mediated immune responses to this protein.

Detailed analysis of the genomic breakpoint junctions in our patient revealed the presence of filler-DNA nucleotides and an inverted repeat flanking the breakpoint junction 5' of *CT45A2*. The repair of chromosomal double-strand breaks (DSBs) occurs by two types of DNA repair pathways: homologous recombination and non-homologous end-joining (NEHJ) [25,26]. The presence of filler-DNA at breakpoints junctions are typical hallmarks of NHEJ [27], and inverted repeats may facilitate the formation of secondary structure intermediates between DNA ends at translocation breakpoints [13]. Moreover, all breakpoint regions were found to be rich in repetitive sequences, particularly LINE/L1 elements and SINE/Alu repeats. Although repetitive sequences may occur near or spanning breakpoint junctions by chance, it is plausible that introns or genomic regions with a high density of repetitive sequences, such as *MLL *intron 9, are more vulnerable to breaking and non-homologous pairing that can lead to gene fusions. However, since the chromosomal breakpoints in our patient were always located outside of these repetitive DNA sequences, it is unlikely that they could be directly involved in the recombination events.

The insertion of 11q23 in Xq26.3 was associated with the deletion of the 3' region of *DDX26B *(encompassing exons 9-16), leading to a premature termination of ddx26b open-reading frame (ORF). The DDX26B [DEAD/H (Asp-Glu-Ala-Asp/His) box polypeptide 26B] protein is a helicase that belongs to the DEAD/DEAH box family of proteins that are considered to be RNA helicases, which have been described to be necessary for, or involved in, many different processes of RNA metabolism [28]. In eukaryotic cells, in particular, these range from transcription to degradation of RNA, and include pre-mRNA splicing, mRNA export, ribosome biogenesis, translation, initiation, and gene expression in organelles [28]. Since our patient is a male, the only functional copy of *DDX26B *is disrupted as a result of the insertion, leading to the absence of the DDX26B protein in the cell or, in alternative, the presence of a truncated protein. Since the particular function of DDX26B in myeloid cells is not known, the phenotypic impact of this abnormality cannot be predicted. The insertion was also associated with loss of the only copy of the *CT45A1 *gene present in the cell. However, since *CT45A1 *expression is restricted to normal testis, the cellular impact of its loss is not expected to be relevant. Similarly, the phenotypic impact, if any, of the insertion in Xq26.3 of 16 additional 11q23 genes (*FXYD6*, *TMPRSS13*, *IL10RA*, *APOO2962.2*, *TMPRSS4*, *SCN4B*, *SCN2B*, *AMICA1*, *MPZL3*, *MPZL2*, *CD3E*, *CD3D*, *CD3G*, *UBE4A*, *ATP5L*, and *AP001267.2*) can also not be predicted.

## Conclusion

We have identified *CT45A2 *as a novel spliced *MLL *fusion partner in a pediatric patient with *de novo *biphenotypic acute leukemia, as a result of a cryptic insertion of 11q23 in Xq26.3. Since *CT45A2 *is the first Cancer/Testis antigen family gene found fused with *MLL *in acute leukemia, future studies addressing its biologic relevance for leukemogenesis are warranted.

## Competing interests

The authors declare that they have no competing interests.

## Authors' contributions

NC designed and performed the research, analyzed the data and drafted the manuscript. CM designed and performed the research, analyzed the data and drafted the manuscript. JS performed the research and analyzed the data. LT, SL, and CC performed the chromosome banding and molecular cytogenetic studies. MP performed sequencing analysis. SB performed RT-PCR analysis. LN clinically assessed the patient. RM and MRT coordinated the study and participated in manuscript writing. All authors read and approved the final manuscript.

## Pre-publication history

The pre-publication history for this paper can be accessed here:

http://www.biomedcentral.com/1471-2407/10/518/prepub
